# Lay perceptions of evidence-based information – a qualitative evaluation of a website for back pain sufferers

**DOI:** 10.1186/1472-6963-6-34

**Published:** 2006-03-15

**Authors:** Claire Glenton, Elin S Nilsen, Benedicte Carlsen

**Affiliations:** 1 Norwegian Knowledge Centre for the Health Services, Pb 7004 St.Olavs Plass, 0130 Oslo, Norway; 2 Stein Rokkan Centre for Social Studies, Nygårdsgt. 5, 5015 Bergen, Norway

## Abstract

**Background:**

In an evidence-informed patient choice the patient has access to research-based information about the effectiveness of health care options and is encouraged to use this information in treatment decisions. This concept has seen growing popularity in recent years. However, we still know relatively little about users' attitudes to the use of research-based information, possibly because people have been unexposed to this type of information. After developing the BackInfo website where the results of Cochrane systematic reviews on the effects of low back pain were adapted and presented to lay users we evaluated how users responded to this information.

**Methods:**

Focus group meetings were held with 18 chronic back pain sufferers, after they had been sent a link to the website before the meetings.

**Results:**

The focus groups suggest that the most important challenges to the use of BackInfo's research-based information are not primarily tied to the comprehension or presentation of the information, but are mainly associated with participants' attitudes towards the credibility of research and researchers, and the applicability of research results to themselves as individuals. Possible explanations for participants' lack of trust in research and their apparent difficulties in applying this research to their own situations include aspects that may be typical for the general public including the media's presentation of research, and a lack of familiarity with and feelings of distance to research evidence. Other aspects may be typical for patient groups with chronic and unclear medical conditions, such as a lack of trust in the health care establishment in general.

**Conclusion:**

In order to enhance the credibility and applicability of research evidence, providers of research-based information could explore a number of possibilities including the use of including personal stories to illustrate the research outcomes.

## Background

### Evidence-informed patient choice

Evidence-informed patient choice "involves providing people with research-based information about the effectiveness of health care options and promoting their involvement in decisions about their treatment" [1]. This concept is based on the assumption that the patient's insight into research-based information will raise the quality of his or her health care decision-making, and is part of a broader assumption that a better public understanding of science in general will enrich society and the individual [2].

In order to make an evidence-informed choice, the patient needs to have access to reliable information about the likely benefits and risks of at least two alternative interventions, which may include the option of no intervention [1]. Ideally, this information should be presented quantitatively and without recommendations.

The growing recognition of patients' desire to be informed [3] is reflected in patient rights' legislation established during the last decade both in Norway and elsewhere [4]. Access to treatment information can be demanded on the grounds that it is the patient's democratic right. In addition, access to information can be seen as a means to better decision making for the individual patient. Researchers evaluating the effect of decision aids providing information about available treatments and their outcomes conclude that they may "improve knowledge and realistic expectations; enhance active participation in decision making; lower decisional conflict; decrease the proportion of people remaining undecided, and improve agreement between values and choice." [5].

Patients are, however, rarely offered reliable information about the effects of health care treatments. A study of four English-language government health portals concluded that these portals very rarely led to specific information about the effect of treatments [6]. In addition, existing patient information has been described as unreliable [7]; medico-centred as opposed to patient-centred [8]; and often consisting of promotional materials for commercial interests [9].

In order to ease access to reliable research evidence, organisations such as The Cochrane Collaboration collects and presents information about the effects of health care interventions in systematic reviews [10]. However, these research summaries are largely inaccessible to lay people because they can be hard to find and contain large amounts of information, and because of medical and research jargon. In addition, the research that forms the basis for these summaries often lacks information about adverse effects, and sometimes covers treatments and outcomes that are convenient to the researcher rather than those that are of relevance to patients [11].

We know little about lay people's attitudes towards this type of information, possibly because of this lack of exposure to research-based information about the effects of health care. In the past, research into the public's perceptions of science has been characterised by a view of the public as "a homogenous mass" and "has drawn upon some notion of the typical citizen" [2]. More recently, however, researchers have seen the public's perception of science as a response to the specific context individual members exist within and the context in which scientific information reaches them [2].

The research-based patient information that has been developed, including a growing number of treatment decision aids, have tended to address individuals facing decisions about conditions such as prostate cancer, breast cancer or pregnancy [5]. For these conditions there exists a high level of agreement about the nature of the condition and the range of treatment alternatives. Decisions are expected to be made within a relatively limited time frame, and the choice of one alternative often precludes the choice of others.

For a number of other illnesses, such as back pain, there is little agreement about either the cause of the disease or appropriate treatments. For chronic back pain sufferers, the lack of a medical diagnosis or a visible disability may lead to experiences of delegitimisation and a fear that the reality of their pain is being questioned [12]. Chronic back pain sufferers are rarely presented with a standard set of treatment choices from which they can choose. Instead, they are often left to search for themselves among a variety of treatments offered by health carers with both alternative and conventional backgrounds. In Norway, where most conventional healthcare, including surgery, medication and physiotherapy, is offered through the public health services, back pain sufferers frequently turn to alternative therapists, such as osteopaths and naprapaths, outside the system. A lack of effective treatments also implies that the decision-making situation is ongoing as the condition often persists and sufferers continue to search for strategies that can help them manage their pain. And as the choice of one alternative rarely precludes the choice of others, back pain sufferers may attempt a series of different treatments over time.

### Developing Backinfo

In this context, we set out to develop Backinfo [13], an Internet-based information service for Norwegian back pain sufferers that aims to present research-based information about the effects of back pain treatment. Backinfo is a collaboration between the Norwegian Back Pain Association, the Cochrane Collaboration Back Group, and the Norwegian Health Services Research Centre. Like most other research institutions in Norway, the Research Centre is defined as a scientifically independent institution but is financed by the Norwegian government.

In order to increase the relevance of the information service, one of the authors carried out a preliminary study of information needs among back pain sufferers [14]. We also carried out user tests and focus groups meetings at different stages of BackInfo's development [15]. As the preliminary study emphasised the need for information about the emotional, financial and social aspects of back pain as well as its clinical aspects, we developed information about welfare benefits and patient rights; and included back pain sufferers' personal stories collected and presented by a journalist. This information was presented separately from the treatment information.

Information about treatment for back pain was made up of research-based information about the effect and possible side effects of 24 different treatments [see Additional files 1 and 2]. In addition, information was provided about treatment accessibility in Norway as well as what to expect before, during and after treatment. Users could find the information that most suited their diagnosis through a search engine. Information about treatment effect was taken from Cochrane reviews but was substantially modified based on the results of pilot testing and guidelines and research about the presentation of health care information to lay people. We have described these processes elsewhere [15]. In short, we systematically extracted data about comparisons, outcomes and measurement time points and presented information about the effect of the treatment and its control in a standardised format. Words and expressions that were difficult to translate into laymen's terms were linked to a glossary. We also presented information about the number of trials and participants that the information was based on.

### The aim of the study

The aim of the study was to investigate back pain sufferers' responses to BackInfo with a particular focus on their understanding of and attitudes towards the research-based treatment information.

## Method

We used focus groups with back pain sufferers to collect data. The presentation of research results to health care users is a relatively new phenomenon in Norway and elsewhere, and BackInfo's contents may be difficult or unfamiliar to its users. Focus groups may show participants that other group members also find the topic difficult and that this is "expected" and "allowed" [16], encouraging "a shift from personal, self blaming psychological explanations (...) to the exploration of structural solutions" [17]. We recruited participants through advertisements on the Back Pain Association website and the BackInfo website, through contacting members of the Back Pain Association, and through flyers at GP offices, hospitals, and chiropractor practices. Participants were recruited if they had easy access to the Internet and if they had back pain themselves or were carers or close family members of a back pain sufferer. To encourage participation, we offered participants a gift voucher.

We held focus group meetings with four groups of back pain sufferers. When the aim of a focus group is to discuss a few topics in relative depth, five or six participants in each group may be optimal (Bojlén and Lunde 1995). Anticipating subject loss, we recruited seven participants per group. Between three and five participants per group, a total of 18 participants, turned up for the meetings. While this number was lower than expected, the data collected from the four groups was similar enough for us to conclude that a point of data saturation had been reached, and no more focus groups were organised. The participants comprised ten women and eight men between 22 and 71 years old. Two-thirds were between 40 and 55 years old, and two-thirds had received some level of formal education or training after high school. One participant was the mother of a chronic back pain sufferer. The others were all chronic back pain sufferers. Twelve participants had had back pain for more than ten years and four had had back pain for between three and ten years. Participants had often tried many of the treatments described in BackInfo.

For reasons of convenience, the focus groups were led by two of the researchers who were responsible for the development of the website. One moderator (CG) led the focus group meetings while one observer (ESN) took notes. Ethical approval for the study was obtained from the local Regional Committee for Medical Research Ethics. Before the focus group meetings, informants were assured of full anonymity and were asked to sign a consent form. Informants were also told that the moderator and the observer were researchers but had no medical background.

The focus group meetings took place in meeting rooms at the research institute, and lasted for an hour and a half to two hours. Participants were sent the website link two weeks before the focus group meetings and were asked to go through the website, paying particular attention to the section with information about the effect of treatments. In addition to an open discussion about the participants' likes and dislikes about the site, we organised the focus group meetings according to a topic guide [See Additional file 3]. Each focus group meeting was audio taped and transcribed.

### Data analysis

The two researchers who led the focus groups also carried out the analysis of the data. In order to counteract possible biases that our involvement in the development of Backinfo might represent we also included a third person (BC), who had no connection with the project, to analyse the data. The framework analysis approach [18], involving five key stages of analysis: familiarization with the data; identification of a thematic framework; indexing; charting; and finally mapping and interpretation, was used to analyse the data from the focus groups. Each researcher independently carried out the first three stages, where themes were identified and data was coded. The researchers then met to discuss and agree upon a common thematic framework, and re-coding was carried out where necessary. All three researchers collaborated in the final two stages, where themes were charted, mapped and interpreted.

Quotes that are used in the article were chosen because they expressed common experiences, attitudes or topics or because they showed the breadth of experiences seen in this group. The informants have been given fictitious names in order to protect their identity.

## Results

Much of the general feedback to BackInfo was positive. People appreciated the fact that different types of information were available in one place. Participants saw the main topics of the website as relevant, although some of them complained that the information was mainly targeted towards the most common age groups and diagnoses. Many participants also complained that information about a number of complementary and alternative treatments were missing from the website.

### Differing views of amount of information and language

Most people used the search engine to find information about the effect of treatments for their particular diagnosis. These searches often resulted in a list of ten to twenty links, each concerning a separate treatment. Participants reacted differently to the lists, some appreciating the amount of choice, others finding the list daunting:

Researcher: Do you have any thoughts about this list?

Karin: It's very long, isn't it?

Olav: Yes, but it has to be!

Ellen: It could have been even longer (as far as I'm concerned)!

Several participants emphasised that they were highly motivated to read large amounts of information:

Lisa: *But if you're looking for information then you want to find some information! That's our problem, that we don't find it. Then it's better to have a proper description, that there's something there*....

Astrid: *You have the time to read it, after all!*

Lisa: *Otherwise you wouldn't be looking for information!*

Astrid: *When you search in the Internet you jump from room to room and time flies without you realizing it. When there's something you're searching for, you read the information that's of interest. So it's not too much to read twenty lines per theme!*

Other participants pointed out that they were just too tired to take in large amounts of information:

Greta: If your back really hurts and you start looking and you see a whole site, then you think "Oh God, I'll be dead before I get through all this!"

For people in these situations, participants suggested a list of "*Top Ten Tips*" describing "*how to move on when you've hit the wall"*. Participants also suggested presenting the information at different levels of detail based on the amount of experience with back pain and back treatments they had.

Some participants thought that the language was too difficult, one of them suggesting that "*we should write as if we were talking to a child*". Most participants, however, appeared to understand the information, and comments about the language were mainly tied to the same four or five medical or scientific terms. The term that led to the most comments was "placebo", even though this was linked to the glossary. However, participants did not agree with our suggestion that this term was replaced with the term "dummy medicine". Instead, they wanted us to use both terms, pointing to the need to familiarise themselves with the words used by their doctors and in their medical records:

Elsa: *I read about TENS and then these words popped up again that should have been written in Norwegian. (Like "placebo".)*

*Researcher: I suppose we could drop that term and just write "dummy medicine"*.

*Lisa: On the other hand, these are the sorts of words that get used when you go and get treatment*...*. It's nice that they get explained. Because when you go to hospital and go to get treatment and stuff then it's those foreign words that get used, and then it's good to know what they mean*.

### Attitudes to research-based information

Although participants did seem to understand the information, they varied in the extent to which they thought they would use it in decision-making. Participants described how they often made treatment decisions in a context of great pain and despair. Under such circumstances, they often had little energy to seek out written information and were sometimes too desperate to care what the research might have to say. Instead, they often gathered information about treatments through the personal anecdotes of friends and neighbours, and, in most cases, this experience-based information was considered to be more relevant than the evidence-based information:

*Jon: This is a really nice site when you're healthy and have some energy. Then you can sort of say "Oh, this works! (..) I'll try that." But when you're very low down, what you have the energy to read is stories from real life, like you have here. How they feel, which treatments they had tried, what happened in practice*.

Researcher: If you had heard that the neighbour had had a [successful] treatment and you went to BackInfo and it said that this treatment had no effect, what would you do then?

*Karin: I would drop that website and listen to the neighbour*.

Some participants did agree that access to the sort of information offered through BackInfo might help them decide which treatments to try first, particularly if it was combined with information about other factors:

*Andreas: When the research says that a treatment doesn't work that well, you might not try it because it's also expensive or has too many side effects*.

Other participants thought that they would be keener to use research-based information if they were facing a decision about a treatment that they perceived to be risky:

Lisa: I'm critical if they start cutting you. Then I really look into it. If they start cutting you or medication or something. But as far as other treatments are concerned, exercise and stuff (...), then I know that it's not dangerous, so then I think, "Let's give it a try."

Several participants also expressed a lack of trust towards research and researchers in general. This lack of trust appeared to be tied to the fact that research results changed over time and that researchers sometimes disagreed among themselves. Some participants also suspected that the research results could be constructed to serve particular interests:

*Ellen: I think, personally that these studies..., well, maybe in two years' time they'll come along with studies that come to different... So I think that ...., I think it's important, but you shouldn't take it too (seriously)*.

*Richard: So a group of researchers have come to the conclusion that (massage) doesn't have much effect. But you can go out on the street and get hold of some other researchers that can prove the exact opposite. (...) We wouldn't have masseuses and acupuncturists that work year in and year out if it didn't help! And it does help back pain patients, I know that from loads of people I've talked to. You can always find researchers who can give you what you want (....)*.

Some participants described the information in BackInfo as impersonal and cold. While some saw this as a negative trait, one participant found these same attributes to be reassuring:

Karin: *I think it's a typical research site, lifeless, there's no warmth. That was my first impression anyway*.

*Harald: But that type of argument, it reminds me of people who want to go to friendly doctors because they think it's so tiresome to go to arrogant doctors. I'm not that worried about..., that is, what's important for me is that I get the right treatment, or in this case, the right information. Whether it's warm and friendly, well, that can be seductive; it can suddenly make you think that everything they write about is good when it's actually all nonsense*.....

The degree to which people were willing to accept and use the research also seemed to depend somewhat on the degree to which this research tallied with their own personal experiences:

Elisabeth: Well, I didn't think there was much point in (that treatment), and if I can get confirmation that it doesn't really give that much effect and this confirms what I myself believe, that's fine. Getting my own opinion confirmed is always nice!

### Research as part of the public health system

Most participants had not noticed who was responsible for the site, but assumed when asked that we were somehow connected to the government and the public health services. A number of quotes suggest that participants did not always make very clear distinctions between researchers, health professionals and the public health services:

*Greta: I don't believe much in research..... (...) When I think of that discussion that there's been with that German doctor, he's done loads of operations, and he's very disputed, and in Norway they wouldn't approve it. And then..., lots of research comes along and says this and that, so I don't always believe the research, because there are so many doctors that are against things while others are for ..... There's this kind of ... professional disagreement, and I'm a bit afraid of that *...

Several participants also assumed that we had particular attitudes about complementary and alternative therapies. One participant was disappointed by the fact that our list of contributors only included "traditional" health professionals such as physiotherapists and doctors. A number of participants suspected that our lack of information about several alternative treatments reflected a bias towards such treatments and that the information that we did provide about these treatments was also biased:

Researcher: When you look at who is responsible for BackInfo, what do you think? Would you trust this information?

Richard: Well, a lot of doctors don't like alternative (medicine) and of course they have their equals in the public health services. I've gone to a doctor who told me that I would be risking my life going to acupuncture. He tells me that I'm wasting my money and that I risk harming myself. (...)

Researcher: So you think that the public health services are biased against alternative medicine?

Richard: Yes, of course they are. (...) There are loads of countries where they've come much further. In the USA they have (acupuncture) in hospitals. (...)

Researcher: Do the rest of you think that we're less likely to say something positive about alternative medicine?

*Eva: I've thought about that! Because getting alternative treatment from the government, from the municipality, it sure wasn't easy*.

*Jon: We back pain patients, we start out having faith in government systems, it doesn't matter which system it is. And gradually, when the system doesn't manage to help you while you sit there and die, then you have to try something else. And when it turns out that the alternative therapy that the system tells you doesn't work actually does work, then it's completely uninteresting for the person in question to discuss whether it has an effect or a placebo effect, whether it's healing or some medicine that falls from the sky, or whether it's some sort of counter-reaction, as long as you get back to work*.

The above statement also illustrates how a question about BackInfo is answered with reference to both the public health services and the government. This apparent lack of distinction may also explain the anger expressed by one participant at BackInfo's presentation of treatments offered through the public health services but that have little effect:

*Eva: The thing is, I get quite depressed about the difference being so small. There were four here and five there.... I got quite preoccupied with all these types of treatment that didn't seem to be worth the bother. And I've tried loads of them! And then I started to think about how I've used all that money, I've paid all those doctors, I've bought all those gadgets, one after the other, and I've thought each time that "this time it'll be better!" And I've thought, sort of, "it must be me there's something wrong with". It's been a really tiresome process, I've just got worse! And now I'm thinking that it's no wonder when I see all this (information). It probably wasn't the right thing for me anyway*.

Researcher: Are you saying that it was good to find out that it wasn't you there was something wrong with?

*Eva: No, no, no! Actually, it makes me quite angry! "What can you believe in?" I think*.

Researcher: So it annoys you that the website describes loads of treatments that don't have any effect?

Eva: Yes! I wonder what the point of it is. Why are all these treatments here if they don't work anyway?

### The relevance of research results to individual cases

Almost all participants questioned the value of statistical results for themselves as individuals. Participants pointed out that it was not possible to tell whether they would end up in one or the other group. In addition, several participants seemed to feel that the research situation was somehow not quite transferable to "real life":

*Astrid: But even if it says that 41 out of 100 get that result and 70 out of 100 get another result I still can't say that I will get that result. But I think it's been explained well*.

*Karin: But it should be emphasized (on the site) that we're individuals. If something doesn't seem to help when they do it for research it might still help someone. You can't let yourself get stuck*.

In addition to our information about the effect of each treatment, we also offered information on what lies behind the research, including the number of trials and participants that the information was based on. Most participants did not look at this information at all. Some participants explained that they were not interested in this type of information, one participant adding that as he had already accepted the website as "solid", there was no reason to double-check what we had based our results on.

Participants who read this information either before or during the group meetings often reacted to what they considered to be low numbers of trial participants. Where numbers were lower than 100, focus group participants were often of the opinion that little weight could be placed on these results:

Jon: If you go to the information on massage, and see the amount of people, there were 51 participants. You're not even close to getting a relevant answer no matter how you look at it!

*Richard: It's way too bombastic*.

*Eva: It's nothing; it's just not good enough*.

### Participants' response to the personal stories

*Harald: I liked the personal stories a lot. I really like reading about real case histories*.

The mixed responses to the research-based information stand in contrast to the enthusiasm almost all participants showed for the personal stories of back pain sufferers. Two participants said that they were not interested in the stories and one person actively did not want to read them because she had had "bad experiences with negativity among other back pain sufferers". All other participants praised the stories, finding it helpful and interesting to read how other people coped, and finding comfort in the fact that others had the same experiences. One participant saw the stories as making up for the "coldness" of the front page:

*Ellen: I thought about what you were saying about it being a cold front page. I thought that this was very alive. I recognized some of it, and I thought it was a very important part of the information*.

## Discussion

### The accessibility of the research-based information

The focus groups suggest that we have managed to present research-based information in a manner that participants are able to understand. Participants also showed a willingness to learn unfamiliar terms as long as these terms are accompanied by simple explanations. Several participants appreciated having access to large amounts of information, although the amount of information was daunting to others, particularly at certain stages in their condition. One way of addressing this issue could be to develop a short overview of all available treatments for each condition.

A desire to understand medical terminology and to learn about one's condition confirms our preliminary study of information needs among back pain patients [14] and is also seen in other studies of patient information services [19,20]. As many back pain sufferers are dependent on health care and on welfare benefits they may be highly motivated to understand these systems and their terminology. The use of medical terms may also be seen as an indication that the health problem is taken seriously [21], a validation of the sick role that is particularly relevant for many chronic back pain sufferers who fear that the reality of their pain is being questioned [12].

### The credibility of the research-based information

The data suggests that the most important challenges to the use of BackInfo's research-based information are not primarily tied to the comprehension or presentation of the information, but are mainly associated with participants' attitudes towards the credibility of research and researchers, and the applicability of research results to themselves as individuals.

Several participants showed a willingness to use research results in specific situations, for instance if the decision was considered to be a particularly serious one, or if it confirmed what they had already decided. This was also seen in the evaluation of Best Treatments, a UK website that offers research-based information on a number of different health conditions. Here, most participants felt that the site was credible and trustworthy because it was based on research and because it coincided with what they themselves had learned [20].

However, many participants in our study also displayed scepticism and distrust towards research and researchers in general. According to Nelkin, the public's view of science is heavily influenced by what they read in the press, as they "understand science less through direct experience or past education than through the filter of journalistic language and imagery" [22]. Within the scientific world, knowledge is portrayed as cumulative, and research becomes reliable through replication and endorsement by professional colleagues. For journalists, by contrast, established ideas may be "old news". Instead, new research is presented in terms of "breakthroughs", "miracle cures" and "new hope" and focus is given to scientific disputes and fraud [23,24]. For chronic back pain sufferers who may have discovered on a number of occasions that "miracle cures" are in fact often unavailable or unsuccessful, this may have led to an impression of research as untrustworthy.

Participants' scepticism towards research may also be influenced by their view of research and research institutions as an extension of the public health services, and other parts of the health care establishment. While other patient groups may view government and professional organisations as impartial and reputable information sources [25,26], back pain sufferers' experiences of delegitimisation at the hands of different parts of the establishment [12], as well as its failure to offer successful treatments may have led to a lack of faith that also extends to health care research. While participants' dependence on the establishment may motivate them to learn its language, this does not necessarily imply that they trust its message.

The assumption that researchers are part of the health care establishment stands in strong contrast to the self-image of research institutions and organisations such as the Cochrane Collaboration. These tend to view themselves as health care watchdogs, emphasising methodological rigour and avoiding financial conflict of interest to protect their independence vis a vis government, industry and professional interests. This assumption may not simply be a reflection of health care users' lack of knowledge, however, but may instead reflect an awareness that most Norwegian research institutions, including our own, are financed by the government. One way of presenting BackInfo as an independent source of information may be to make it clear to users that the information found on BackInfo may or may not be in keeping with the view and practice of the Norwegian health care system.

Participants' scepticism also appears to be tied to the assumption that BackInfo is biased against alternative medicine. Backinfo's lack of information about alternative treatments does not reflect any bias on our part but is due to a lack of Cochrane reviews on these topics. This lack of information probably does reflect some sort of bias somewhere on the journey between treatment use and the production of systematic reviews. One way of addressing this issue might be for BackInfo to at least include descriptive information about alternative treatments, including how, when and where they are used, to include referees that are alternative therapists, and to present explanations of why information about effect is missing. In addition, alternative therapists could be included as contributors to BackInfo.

The belief that BackInfo is biased against alternative medicine might also be connected to the assumption that we are part of the establishment. This assumption could be tied to the fact that participants were invited to the focus group meetings by a government-financed research institution. We do not know how other users of BackInfo would view the website. Some studies show that while users claim to judge the credibility of a website by its source, they do not necessarily search systematically for information about this source [7]. Instead they may make assumptions based on the site's look and feel [25,26]. BackInfo's "look and feel", including its lack of advertising, it's exclusion of many alternative treatments, and it's inclusion of research-based information about primarily conventional treatments, may point in the direction of a non-commercial, public site. By drawing more attention to our collaboration with the Norwegian Back Pain Association and emphasising the non-governmental and non-commercial status of the Cochrane Collaboration it might also be possible to increase BackInfo's credibility.

### The applicability of the research-based information

Regardless of their degree of trust in research and researchers, most of the participants found it difficult to apply research results to themselves as individuals. Participants' assertion that it is impossible to know whether they will end up in one group or another reflects an uncertainty that is inherent in the nature of medical evidence [27]. However, many participants were also uninterested in using these statistics as an indication of their probability of achieving treatment success.

The public's lack of exposure to research-based information means that they have little experience in transferring this type of information to their own situation. Their lack of interest in using the research results may also be tied to a desire to sustain hope and avoid discouragement. Leydon and colleagues describe how cancer patients see hope as indispensable for survival. For some this meant "avid searching for information, particularly about alternative treatments, but for others it meant limiting searching for or even avoidance of new information" [28]. Access to healthcare information can be seen as empowering to the individual [29] and may help users make treatments decisions. On the other hand, BackInfo shows that many back pain treatments have little documented effect, and the presentation of information that is experienced as discouraging may instead lead to feelings of helplessness and a wish to avoid this type of information.

Chronic back pain sufferers and others who do not face clear choices between two different treatments and who can choose one treatment type without precluding another may also feel less of a need to transfer research statistics to their own situations. Back pain sufferers facing a decision about whether or not to have surgery, on the other hand, may feel a greater need to apply these numbers to themselves as the treatment is considered to be more risky than other treatments.

Several of the quotes also suggest that research is somehow not quite real to participants. While the research is described as "lifeless" and "impersonal", the personal stories are referred to as "stories from real life", "real case histories", "what happens in practice". This view of research as different from "real life" might also explain why participants did not consider 51 trial participants a sufficient number to base a decision on, while these same participants were willing to base a decision on the testimony of one or two friends or neighbours.

### Integrating research-based and experience-based knowledge

Studies of information needs, including our study among back pain sufferers [14] show that information about other peoples' experiences is important to patients and is often used instead of [26,28] or in addition to [30] medical information. Experience-based information can place abstract concepts of disease in the broader context of the illness experience and in the narrow context of the individual. In contrast, the analysis of research data involves the decontextualisation of this data in order to make generalisations [31]. During this process, individual fates and experiences are lifted out of their environments and common traits are merged. Is it possible to increase the applicability and credibility of scientific information by placing research results back into the context of the individual patient?

The media typically achieves this contextualisation by using case histories to illustrate research articles. There is, however, a fear that the presentation of individual cases, particularly when facts are overdramatised and atypical cases are portrayed, may distort the original meaning of the research [23]. Within evidence-based health care, experience-based knowledge is at the very bottom of the evidence hierarchy with regard to research about effect. While experience is looked upon as a legitimate part of the evidence-informed decision [32], too close an association with anecdotal evidence may even be regarded by some as "heresy" [33].

However, the emphasis placed on this type of information by many patients suggests that a closer integration of this type of information could help to personalise the research findings and present them in more meaningful ways [33]. Irwin et al argue that "(i)f scientists are sincere in their desire to communicate more effectively with the rest of society – than this will involve a willingness to engage with alternative worldviews and "knowledges" rather than labelling them in advance as emotive and ignorant" [34].

While BackInfo did include personal stories that we collected in response to our study of information needs, we argued at the time that the evidence-based and experience-based information should be clearly differentiated. The stories were therefore presented separately from the research-based information. By integrating representative personal stories with the research-based information, while at the same time making the user aware of what he or she is reading, it might be possible to influence users' attitudes about the relevance and credibility of the research information. This has, for instance, been attempted in an interactive videodisc program for low back pain patients contemplating surgery where patient interviews are used to exemplify both good and bad outcomes [35]. This type of information could be collected by journalists, or more systematically, by carrying out qualitative interview studies and then presenting purposive samples of these interviews, as has been done for the UK-based DIPEX website of patient experiences [36].

Personal stories from other back pain sufferers might also be used to give users information about coping strategies, a type of information that is particularly helpful for chronic patient groups, and that may be at least as important as treatment information.

## Conclusion

Other research suggests that the provision of information about treatment options and their expected outcomes can lead to better decision making [5]. A number of barriers may, however, prevent particular patient groups from utilising this type of information.

Irwin and Wynne emphasise that the public's acceptance of scientific information is not only a question of "presentation" or "communication" of what are assumed to be already-validated knowledges [37]. The present study suggests that people's willingness to accept and make use of research-based information may also be influenced by the degree in which it is seen as credible and applicable to their own situation.

We have proposed a number of possible explanations for participants' lack of trust in research and their expressed difficulties in applying the results of this research to their own situations. These explanations may be associated with aspects shared with other patient groups, such as the media's presentation of research, and a lack of familiarity with research evidence. Other aspects may be typical for back pain sufferers and other patient groups with chronic and unclear medical conditions, such as a lack of trust in the health care establishment and a lack of clear treatment alternatives. Yet other aspects may be typical for societies such as Norway where conventional health care and research institutions are traditionally government-funded. Comparative studies of different patient groups in different settings could be used to further investigate these issues.

In order to enhance the credibility and applicability of research evidence, we have suggested a number of solutions that providers of research-based information could explore. A solution that might address both the credibility and the applicability of the information is the use of representative personal stories to illustrate the research outcomes.

## Competing interests

The author(s) declare that they have no competing interests.

## Authors' contributions

CG developed the protocol for this study with assistance from ESN and BC. CG recruited participants for the focus groups, developed the interview guide, carried out the focus groups and transcribed the focus groups with assistance from ESN. CG, ESN and BC analysed the focus groups. CG wrote the manuscript and revised it based on comments from BC and ESN. CG will act as guarantor of the paper.

## Pre-publication history

The pre-publication history for this paper can be accessed here:



## Supplementary Material

Additional File 1 "Appendix 1. Treatments presented in BackInfo" Click here for file

Additional File 2 "Appendix 2. "Example of BackInfo information about treatment effect" Click here for file

Additional File 3 "Appendix 3. "Focus group topic guide" Click here for file
