# Supplementary material for: Lay perceptions of evidence-based information – a qualitative evaluation of a website for back pain sufferers
Source: BMC Health Serv Res. 2006 Mar 15;6:34. doi: 10.1186/1472-6963-6-34 (PMC1459152; doi:10.1186/1472-6963-6-34)
Supplement: Additional File 1 — "Appendix 1. Treatments presented in BackInfo" [file 1472-6963-6-34-S1.doc]

**Appendix 1. Treatments presented in BackInfo**

1. Acupuncture
2. Back school
3. Bed rest
4. Bio-psycho-social rehabilitation
5. Cognitive behavioural treatment
6. Disc surgery: microdiscectomy
7. Disc surgery: open discectomy
8. Disc surgery: percutaneous discectomy
9. Disc surgery: chemonucleolysis
10. Disc surgery: Rehabilitation after surgery
11. Exercise
12. Epidural injections
13. Facet injections
14. Lumbar support belts
15. Massage
16. Muscle relaxants: benzodiazepines
17. Muscle relaxants: non-benzodiazepines
18. Muscle relaxants: antispasticity drugs
19. NSAIDs
20. Radiofrequency denervation
21. Radiofrequency thermocoagulation
22. Staying active
23. TENS
24. Treatment in pregnancy: Ozzlo pillows
25. Treatment in pregnancy: water gymnastics
