# Supplementary figures and images for: Lay perceptions of evidence-based information – a qualitative evaluation of a website for back pain sufferers
Source: BMC Health Serv Res. 2006 Mar 15;6:34. doi: 10.1186/1472-6963-6-34 (PMC1459152; doi:10.1186/1472-6963-6-34)

**Appendix 2. Example of BackInfo information about treatment effect**


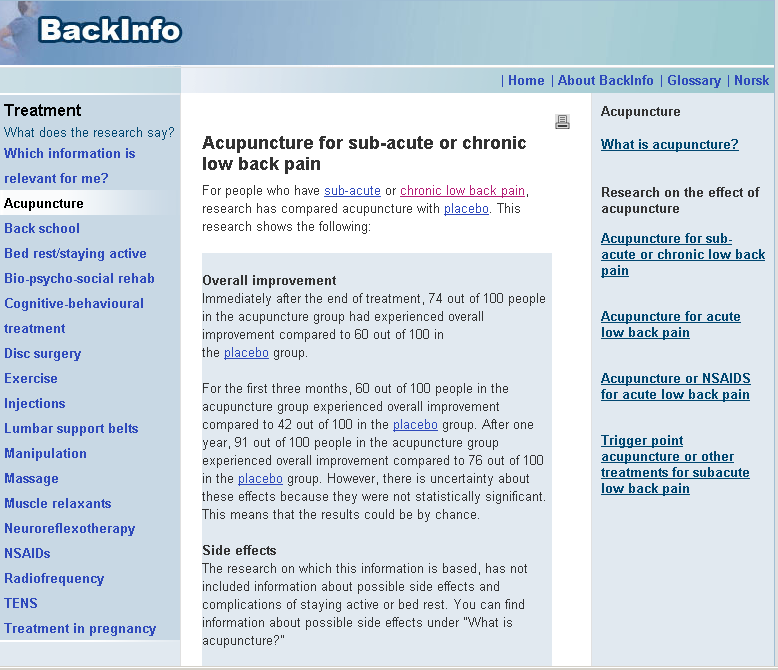


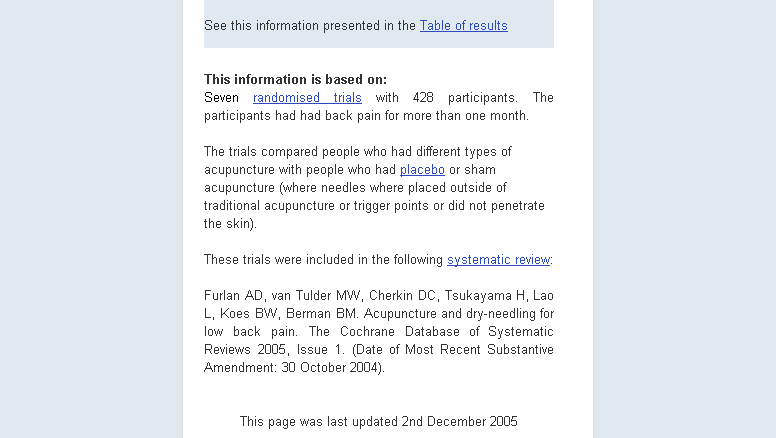

Supplement: Additional File 2 — "Appendix 2. "Example of BackInfo information about treatment effect" [file 1472-6963-6-34-S2.doc]
