# Supplementary material for: Lay perceptions of evidence-based information – a qualitative evaluation of a website for back pain sufferers
Source: BMC Health Serv Res. 2006 Mar 15;6:34. doi: 10.1186/1472-6963-6-34 (PMC1459152; doi:10.1186/1472-6963-6-34)
Supplement: Additional File 3 — "Appendix 3. "Focus group topic guide" [file 1472-6963-6-34-S3.doc]

**Appendix 3. Focus group topic guide**

*(During each question, the relevant part of the website is displayed by projector)*

**Your areas of interest:** What kind of information are you interested in at the moment? Are there particular topics that are relevant or of interest to you right now?

**Front page – Expectations and first impressions:** Think back to when you first opened the web page.

- What did you think you would find?
- Who did you think was responsible for the website?
- Would you trust the information you found here?

**Were first expectations and impressions met?** Did you find information that you were interested in? Were your first impressions met when you used the website or did your impressions change?

**Rules and benefits:** What, if anything, did you like about this section? What, if anything, did you not like about it? Was there any information that you missed? Was there any information that you think could be removed?

**Living with back pain:** What, if anything, did you like about this section? What, if anything, did you not like about it? Was there any information that you missed? Was there any information that you think could be removed?

**Treatment for back pain – general:** Which treatments did you read about? (*Go to treatments that people looked at*.) What, if anything, did you like about this information? What, if anything, did you not like about it? Was there any information that you missed? Was there any information that you think could be removed?

**Treatment for back pain – language:** What did you think of the language? Was it too difficult? Too easy?

**Treatment for back pain – length:** What did you think of the length of the information? Was it too long? Too short?

**Treatment for back pain – information about effect:** Was it difficult or easy to understand this information? Was anything unclear of confusing?

**Treatment for back pain – information about side effects:** Was it difficult or easy to understand this information? Was anything unclear of confusing?

**Treatment for back pain – results tables:** Did you look at the tables? Was it difficult or easy to understand this information? Was anything unclear of confusing? How would you explain the results of these tables to another person?

**Using Backinfo**

- Did you become interested in trying new treatments or perhaps ending current treatments after reading BackInfo? If so, how would you go about doing this?
- Would you bring information from BackInfo along to your doctor?
